# Heterogeneous Wafer Bonding Technology and Thin-Film Transfer Technology-Enabling Platform for the Next Generation Applications beyond 5G

**DOI:** 10.3390/mi12080946

**Published:** 2021-08-11

**Authors:** Zhihao Ren, Jikai Xu, Xianhao Le, Chengkuo Lee

**Affiliations:** 1 Department of Electrical & Computer Engineering, National University of Singapore, 4 Engineering Drive 3, Singapore 117576, Singapore; elerz@nus.edu.sg (Z.R.); e0496474@u.nus.edu (J.X.); elelexia@nus.edu.sg (X.L.); 2 Center for Intelligent Sensors and MEMS (CISM), National University of Singapore, 5 Engineering Drive 1, Singapore 117608, Singapore; 3 National University of Singapore Suzhou Research Institute (NUSRI), Suzhou Industrial Park, Suzhou 215123, China; 4 NUS Graduate School for Integrative Science and Engineering, National University of Singapore, Singapore 117456, Singapore

**Keywords:** heterogeneous integration, wafer bonding, thin-film transfer, System-in-Package (SiP), sensor, 5G, 6G, photonics, power electronics, Internet of Thing (IoT), Artificial Intelligence of Thing (AIoT), wearable electronics

## Abstract

Wafer bonding technology is one of the most effective methods for high-quality thin-film transfer onto different substrates combined with ion implantation processes, laser irradiation, and the removal of the sacrificial layers. In this review, we systematically summarize and introduce applications of the thin films obtained by wafer bonding technology in the fields of electronics, optical devices, on-chip integrated mid-infrared sensors, and wearable sensors. The fabrication of silicon-on-insulator (SOI) wafers based on the Smart Cut^TM^ process, heterogeneous integrations of wide-bandgap semiconductors, infrared materials, and electro-optical crystals via wafer bonding technology for thin-film transfer are orderly presented. Furthermore, device design and fabrication progress based on the platforms mentioned above is highlighted in this work. They demonstrate that the transferred films can satisfy high-performance power electronics, molecular sensors, and high-speed modulators for the next generation applications beyond 5G. Moreover, flexible composite structures prepared by the wafer bonding and de-bonding methods towards wearable electronics are reported. Finally, the outlooks and conclusions about the further development of heterogeneous structures that need to be achieved by the wafer bonding technology are discussed.

## 1. Introduction

Wafer bonding is a popular technology that can integrate two or more kinds of materials into one heterogeneous system. It has been successfully applied to system-in-package (SiP) and fabrication of heterogeneous structures [1,2,3,4,5,6,7]. Traditional wafer bonding technology is mainly based on silicon (Si)-based materials and is oriented to the microelectromechanical system (MEMS) packaging [8,9,10]. Due to the high-quality bonding interface, wafer bonding can satisfy meet the strict requirements of MEMS devices in terms of airtightness, bonding strength, and interface corrosion in extreme environments. Combined with the ion implantation, a Smart Cut^TM^ technology (Soitec, Bernin, France), has been proposed and used for the fabrication of high-quality single-crystal thin film onto different substrates. Correspondingly, the application of wafer bonding technology has been dramatically expanded. Among them, Si-on-insulator (SOI) wafers that have been widely used in various electronic products and photonic devices are one of the most successful commercial cases. The top Si thin film can serve as the functional layer for both electrical and optical designs. With the rapid rise of new energy vehicles, wafer bonding technology of wide-bandgap semiconductors, such as gallium nitride (GaN), silicon carbide (SiC), and gallium oxide (Ga_2_O_3_), for power electronics is gradually being developed and improved [11,12,13]. Due to the advantages of being able to transfer high-quality single-crystal thin film without consideration of lattice mismatch, wafer bonding combined with ion implantation or laser irradiation possess more flexible features in the processing and heterogeneous integration of power chips.

Currently, we are on the cusp of communication technology innovation. The development of the fifth-generation (5G) communication technology and alternative communication technologies beyond 5G will affect the technology landscape of countries worldwide. The communication technologies aim to be a revolutionary leap forward in data rates, latency, massive connectivity, network reliability, and energy efficiency [14,15,16,17]. Therefore, high data-transmission requirements force a rapid improvement in the performance of terminal products. The arrival of the 5G era is inseparable from the development of radio frequency (RF) front-ends for telecom infrastructure, and the core of the RF front-ends are the filters composed of piezoelectric resonators. Piezoelectric resonators with high electromechanical coupling will improve the performance of the RF front-end, e.g., increase in the bandwidth and reduce the loss. Among the piezoelectric resonators using various structures and piezoelectric materials, the piezoelectric resonator based on lithium niobate (LiNbO_3_) thin film has a significantly high electromechanical coupling. However, it is currently not possible to achieve the preparation of LiNbO_3_ film on the substrate/insulator through conventional deposition processes. Exploiting the transfer technique to move a slice of the bulk LiNbO_3_ and bond to a carrier substrate is the most common and effective method [18,19]. Moving into the era of 5G and beyond 5G, among diversified components and devices that integrate on the chip in the wafer fabrication process, the thin-film LiNbO_3_ electro-optic modulator has shown outstanding advantages in terms of the speed of information transmission and bandwidth modulation [20]. As for the fabrication of the LiNbO_3_-on-insulator (LNOI) platform, wafer bonding provides effective technical support for the on-chip heterogeneous integration of LiNbO_3_ thin film. This idea has been confirmed to be feasible by designing and preparing high-speed modulators on LNOI wafers, which are fabricated using the wafer direct bonding technology to peel off the single-crystal thin film from the bulk LiNbO_3_ crystals [21,22]. This will be beneficial for the next generation applications beyond 5G communication.

In addition to the above mentioned on-chip integrated applications, thin-film transfer relying on the combination of bonding and de-bonding methods can also be used to fabricate wearable electronics. With the continuous improvement of life quality for human beings, wearable sensors and human-machine interfaces have been considered essential for future multi-functional sensors related to human health [23,24,25,26,27,28]. However, flexible substrates, such as polydimethylsiloxane (PDMS) and polyimide (PI) cannot tolerate high-temperature treatment. Therefore, functional thin films are difficult to prepare on the flexible substrates without damages by sputtering, evaporation, and deposition methods. One of the most effective solutions is to deposit the targeted film onto the Si substrate cladding with hundreds of nanometers of silicon dioxide (SiO_2_). Then, thin films can be transferred onto flexible substrates using the combination of sacrificial removal processes by hydrofluoric acid solution and hot-press bonding processes. Due to the bonding and de-bonding processes compatible with the complementary metal-oxide-semiconductor (CMOS) technology can provide large-scale and stable substrates for subsequent device fabrication. Meanwhile, in the era of big data and artificial intelligence, next-generation wearable sensors will enable human beings to interact with other objects in more and more applications. Driven by such trends, the Internet of Things (IoTs) and artificial intelligence of things (AIoTs) are indispensable considerations for future sensor design and development [29].

**Figure 1:**
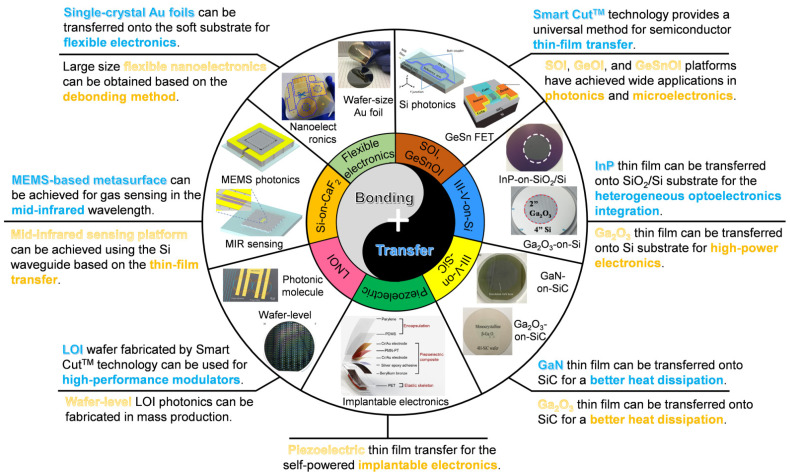
Schematic illustration for the classification of thin-film transfer via bonding methods and their applications in photonics, power electronics, and flexible electronics.

In this review, we summarized the high-quality thin films fabricated using wafer bonding technologies and transferred methods in different fields. Divided by materials, we reviewed electronics devices, optoelectronics devices, on-chip integrated mid-infrared sensors, and wearable sensors for communication and sensing applications. The schematic diagram of the article structure is shown in Figure 1. Firstly, we introduce the X-on-insulator platforms fabricated by the Smart Cut^TM^ process, including wafer bonding and ion implantation processes. Additionally, applications of different platforms in the on-chip integration are presented. Secondly, the different wide-bandgap semiconductor thin-film transfer progress based on the wafer bonding methods for high-power electronics is reported. Thirdly, achievements of LiNbO_3_ thin-film transfer for high-speed electro-optical modulators towards the next generation of communication technology are summarized. Fourthly, film transfer of infrared materials and their applications in molecular sensors are systematically introduced. Finally, we emphasize the methods on the functional thin-film transfer onto flexible substrates for the next generation of wearable sensors, which are related to human health monitoring.

## 2. Fabrication of Si- and Ge-Based Thin Film-on-Insulators via Wafer Bonding Method

Semiconductors are fundamental for all kinds of photonic and electronic components. They are the basic platform for device design and fabrication. Si- and Ge-based materials are the most popular among numerous semiconductors due to the advances in mature fabrication, low cost, and mass production [30,31]. Therefore, most complementary-metal-oxide-semiconductor (CMOS) process development depends on them. However, the device layers used for active and passive devices are usually nanometer- or micrometer-level in thickness. To avoid signal crosstalk or element diffusion, the insert barrier layer is indispensable between the adjacent device layers. Therefore, the fabrication of Si- or Ge-based thin film on insulators is urgently needed to solve. Although such films can be grown on specific substrates via sputtering, chemical vapor deposition (CVD), plasma-enhanced chemical vapor deposition (PECVD), and so on, amorphous or polycrystalline crystal structures are challenging to maximize the excellent material performance. A wafer bonding method combined with ion implantation (known as Smart Cut^TM^ technology) has been developed [32,33]. This technology makes it possible to transfer a thin layer from a single-crystal donor substrate to the supporting wafer, overcoming physical limitations and changing the face of the substrate industry.

### 2.1. Smart Cut^TM^ Technology for the Fabrication of Si-on-Insulator and Its Applications

**Figure 2:**
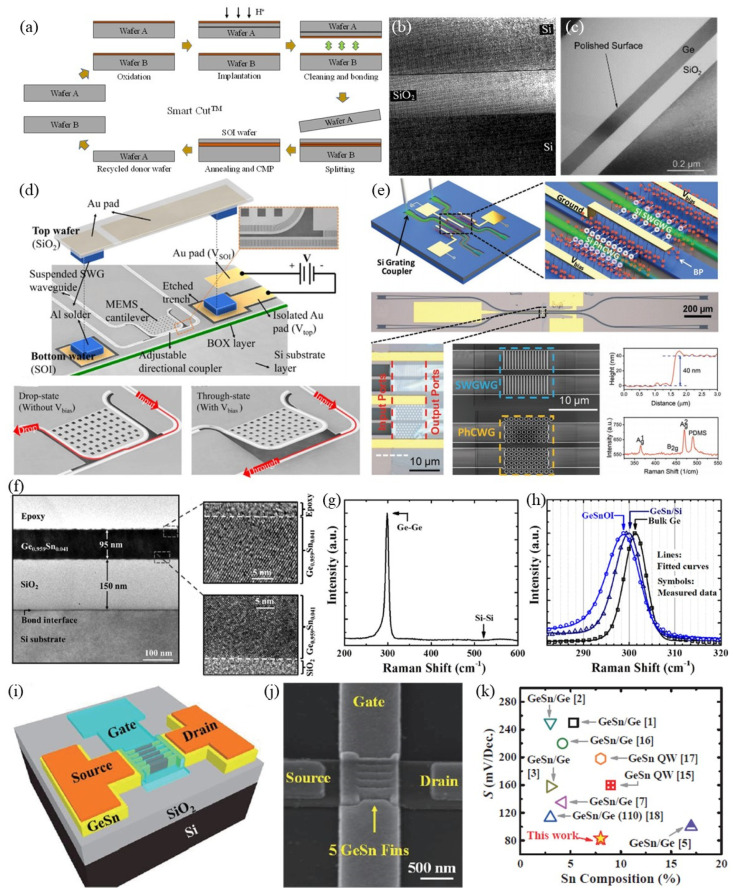
Smart Cut^TM^ process for the fabrication of SOI, GeOI, and GeSnOI, as well as their applications in photonics and electronics. (**a**) Flow chart of the Smart Cut^TM^ process for the thin-film transfer. (**b**) TEM image of the transferred SOI interface [34]. (**c**) Transferred GeOI interface observed by TEM [40]. (**d**) A typical application of SOI in MEMS-based tunable waveguide coupler [53]. (**e**) A traditional application of SOI wafer in MIR slow light waveguide photodetector [54]. (**f**) Interfacial characterization for the GeSnOI wafer fabricated by the direct bonding method [55]. (**g**) Interface studies for the bonded GeSnOI wafer [55]. (**h**) Strain calculation for the GeSnOI wafer based on the comparison of Raman spectra [55]. (**i**–**k**) Application of the bonded GeSnOI wafer in the GeSn FinFET [56]. Reprinted with permission from ref. [34]. Copyright 1997 Japan Society of Applied Physics. Reprinted with permission from ref. [40]. Copyright 2004 Springer. Reprinted with permission from ref. [53]. Copyright 2020 The Optical Society. Reprinted with permission from ref. [54]. Copyright 2020 Wiley-VCH. Reprinted with permission from ref. [55]. Copyright 2016 American Institute of Physics.

Today, most industry-leading Si-on-insulator (SOI) wafers destined for chip manufacturing are made by Smart Cut^TM^ technology. The standard Smart Cut process for the SOI fabrication is shown in Figure 2a. Surface oxidation is first applied to both cleaned Si wafers. One of them is used as the supporting substrate, which is used for the thin-film donor wafer. Then, hydrogen ion (H^+^) implantation creates a destroyed layer (i.e., bubble layer) on the subsurface. There are several advantages to implant the light element (i.e., H^+^) into the donor wafers. The transferred layer can be engineered with no defects. By controlling the implantation energy of H^+^, the thickness of the transferred layer can be determined with a high degree of precision. After finishing the ion implantation, the oxidized wafers are bonded together via an activated bonding method. Then, an annealing process is performed on the bonded pairs. On the one hand, the donor wafer will be completely cracked from the bubble layer, making the thin film peel off and remain on the supporting wafer. On the other hand, high-temperature treatment will be beneficial for strengthening the bonding interface between the thin film and supporting substrate. Finally, chemical-mechanical polishing (CMP) is used for flattening and smoothing surfaces to satisfy practical production requirements. For the donor wafers, substrates can be refreshed and reused after each layer transfer operation. The cross-section image of the fabricated SOI wafer is shown in Figure 2b [34]. A dense and sharp interface without any defects can be observed by transmission electron microscopy (TEM), proofing the advantages of Smart Cut technology. We should note that wafer bonding is suitable for a few kinds of materials and a wide range of temperatures, opening the way to enable any thin-film materials onto other substrates while maintaining initial crystallographic properties. For example, Ge is another basic material in the electronics industry [35,36,37,38,39]. GeOI is also an essential platform for high-performance field-effect transistors (FET). Based on the Smart Cut technology, single-crystal Ge thin film can be transferred to other substrates. TEM study indicates that the bonding interface is void-free, as shown in Figure 2c, confirming this process can be applied to the thin-film transfer beyond Si material [40]. Besides, Smart Cut technology is based on standard semiconductor equipment and allows different sizes of products. The thickness of top thin-film layers and buried oxide layers can also be adjusted with substantial flexibility. Moreover, the excellent quality in terms of uniformity, bonding interfaces, and precise control of thickness variability cannot be achieved by other methods. This technology also provides new opportunities for growing and emerging fields, such as sensors, energy harvesting, flexibles electronics, photonics, and MEMS, etc. [41,42,43,44,45,46,47,48,49,50,51,52].

Recently, a typical multifunctional photonic switch on the SOI platform operating in the mid-infrared (MIR) wavelength range (from 3.85 μm to 4.05 μm) has been reported [53]. They used suspended waveguides with sub-wavelength cladding and MEMS to tune the waveguide coupler. The schematic diagram of the proposed device is shown in Figure 2d. SiO_2_ wafer and SOI wafer are the upper and lower layers of the device, respectively. Microstructures patterned on the two parts are fabricated separately and assembled via the flip-chip bonding method. The optical waveguide couplers are prepared on the bottom SOI wafer, including suspended subwavelength grating (SWG) waveguides, grating couplers, and a tunable waveguide coupler with a MEMS cantilever. To make the MEMS cantilever move up and down, a single-side cladding for the suspended SWG waveguide is used to replace double-side cladding in the cantilever region. Leveraging the advantage of this change, the movement of the MEMS cantilever and optical signal transmission along the waveguide can be simultaneously realized with less crosstalk. The Au pad is fabricated on the top SiO_2_ wafer and covers the cantilever region. Al solders are used for the intermediate bonding of the top SiO_2_ wafer and bottom SOI wafer and the spacers and electrical interconnections. The MEMS cantilever can be actuated by applying a voltage between the top and bottom electrodes. The light propagates in the waveguide and couples from the input-port to drop-port in the lack of voltage bias. Correspondingly, the power-splitting ratio can be continuously tuned in the range of 0% and 100%. Based on the advances of the SOI platform and design, an optical attenuator with 25 dB depth using DC voltage actuation has been realized. With the actuation of AC voltage, the 1 × 2 optical switches with a response time of 8.9 μs and −3 dB bandwidth up to 127 kHz has been demonstrated.

Due to the excellent infrared properties of Si, another important application for the SOI platform is used for the photodetectors combining with two-dimension materials (e.g., black phosphorus, BP) [54]. A design for the shared-BP photonic system composed of photonic crystal waveguides (PhCWCs) with a slow light effect for the responsivity enhancement has been proposed. The schematic diagram and physical images of the device structures are shown in Figure 2e. A PhCWC and a subwavelength grating waveguide (SWGWG) with equal length are designed to highlight the slow-light enhancement. The BP covers the waveguide surfaces and is shared by the PhCWC and SWGWG at the same time. In the 3.8 μm wavelength, a responsive enhancement by more than tenfold in BP photodetector has been demonstrated. Such a slow light waveguide photodetector shows great potential for the on-chip MIR system in a wide range of applications, including environmental monitoring, industrial process control, and medical diagnostics.

### 2.2. GeSn-on-Insulator Platform Fabrication and Its Applications in FETs

Germanium-tin (Ge_1−x_Sn_x_) alloy is a famous Group IV semiconductor for potential applications in both electronics and photonics [57,58,59,60,61]. By controlling the content of Sn, the bandgap of Ge_1−x_Sn_x_ can be tuned. The combination of Group IV-based optical components with high-speed CMOS circuits can be desirable in future integrated chips. Therefore, the high-quality Ge_1−x_Sn_x_ layer on the insulator is an essential platform for monolithic integration. Currently, Ge_1−x_Sn_x_ thin film with a surface roughness of 0.528 nm can be grown on the (001)-oriented Si substrate using a molecular beam epitaxy (MBE) system. To fabricate the Ge_1−x_Sn_x_ on insulator structure, a 500 nm-thickness SiO_2_ layer is deposited on the Ge_1−x_Sn_x_ using the PECVD. To make the SiO_2_ denser, a 350 °C thermal treatment for 7 h in an N_2_ environment is conducted. Subsequently, the CMP process is performed on the SiO_2_ surface to reduce the surface roughness. The root-mean-square (RMS) value smaller than 0.2 nm has been achieved, which can satisfy the requirement for wafer direct bonding [62,63,64,65,66,67,68,69,70,71,72,73,74]. Then, both SiO_2_/Ge_1−x_Sn_x_/Si and Si supporting wafer surfaces are activated by the O_2_ plasma for 15 s and contacted at room temperature. A post-annealing at 300 °C in an N_2_ environment is carried out to further enhance the bonding strength. Finally, the top Si is removed by soaking the bonded pair into tetramethylammonium hydroxide (TMAH) solution with an 80 °C heating treatment, while the bottom Si supporting wafer is spin-coated by a protective layer to avoid being etched. The cross-sectional TEM image of the obtained GeSnOI is shown in Figure 2f [55]. The bonding interface is flat and void-free. The thickness of the Ge_1−x_Sn_x_ layer is 95 nm which is the same as the one grown by MBE, revealing the high etching selectivity of TMAH for the Si and Ge_1−x_Sn_x_.

To characterize the chemical bonds and strain of the fabricated GeSnOI wafers, Raman spectra are used for the component analysis, as shown in Figure 2g,h [55]. It can be seen that a peak assign to be Ge-Ge vibration mode at 300 cm^−1^ is appeared, while the peak at 520 cm^−1^ is contributed by the Si-Si vibration mode nonexistent. This indicates that the Si has been entirely removed by the TMAH solution, as presented in Figure 2g. By comparing the Raman shifts among the GeSnOI, GeSn/Si, and bulk Ge wafers, a compressive strain of GeSnOI substrate is calculated to be 0.10% which is smaller than 0.26% in the original Ge_1−x_Sn_x_ substrate, as shown in Figure 2h. The high-quality transferred GeSn thin film will bring enormous benefits for the fabrication of high-performance GeSn PFET. Based on the GeSnOI platform, a GeSn FinFET has been reported. The schematic diagram of the device structure is shown in Figure 2i [56]. The scanning electron microscopy (SEM) image of the device is shown in Figure 2j [56]. The channel lengths and fin width are down to 50 nm and 20 nm, respectively. Compared with other reported GeSn p-FETs, record-low *S* of 79 mV/decade and record-high *G_m,int_* of 807 μs/m (*V_DS_* of −0.5 V), as well as highest *G_m,int_/S_sat_* are realized. The result of the comparison for S in GeSn p-FET is shown in Figure 2k [56]. These results are the world’s first GeSn FinFETs demonstrated on a GeSnOI substrate with excellent electrical performance. The enablements mentioned above are attributed to the high-quality GeSnOI heterogeneous structure obtained by the layer transfer. The encouraging achievements show that the GeSnOI platform holds promise for future advanced applications.

## 3. Heterogeneous Bonding for III-V and Wide Bandgap Semiconductor Thin-Film Transfer onto Si Substrate

As mentioned above, Si-based CMOS is one of the most fundamental components in semiconductor products. Driven by Moore’s Law in the past year, device miniaturization and multifunction are the goals of chip development and pursuit. As the feature size continues to scale down, many advantages, such as improved operation speed, reduced power consumption, and production cost, gradually emerges [75,76,77]. Unfortunately, device reliability issues, such as short channel effects and random fluctuations, become more severe when the size approaches the limitation of Si-CMOS [78]. Besides, the substantial increase in the cost of lithography and etching processes makes the price of devices no longer dominant. The device design and its performance will be more and more materials-driven, along with the benefits of scaling subside. Therefore, the development of heterogeneous material systems compatible with the Si-CMOS platform can be the next generation of semiconductor technologies to break through the bottleneck of Si-CMOS scaling. Among numerous semiconductors, III-V compound materials, such as InP, GaAs, and GaN, receive widespread attention due to the excellent electrical properties and high-electron-mobility in transistors [79,80,81,82]. Compared with Si, most III-V compounds have a direct bandgap, making them can be used as light-emitting diodes (LEDs) and lasers [83,84,85,86,87]. The combination of III-V materials with the Si-CMOS platform can be used as a hybrid solution for the on-chip integration of Si-based photonics. Although they can directly grow on the Si substrate via epitaxial growth, many issues, such as significant mismatch in the lattice and coefficient of thermal expansion (CTE), still need to solve and improve. In contrast, direct wafer bonding is an efficient method to join hetero/homogeneous materials into one composite at low temperatures. Based on the Smart Cut^TM^ technology, it is possible to integrate thin film of III-V compounds onto the Si-CMOS platform. In addition to compound semiconductors, wide-bandgap materials, such as Ga_2_O_3_, are also popular in optoelectronic devices.

### 3.1. InP Thin-Film Transfer Based on the Modified Smart Cut^TM^ Technology

**Figure 3:**
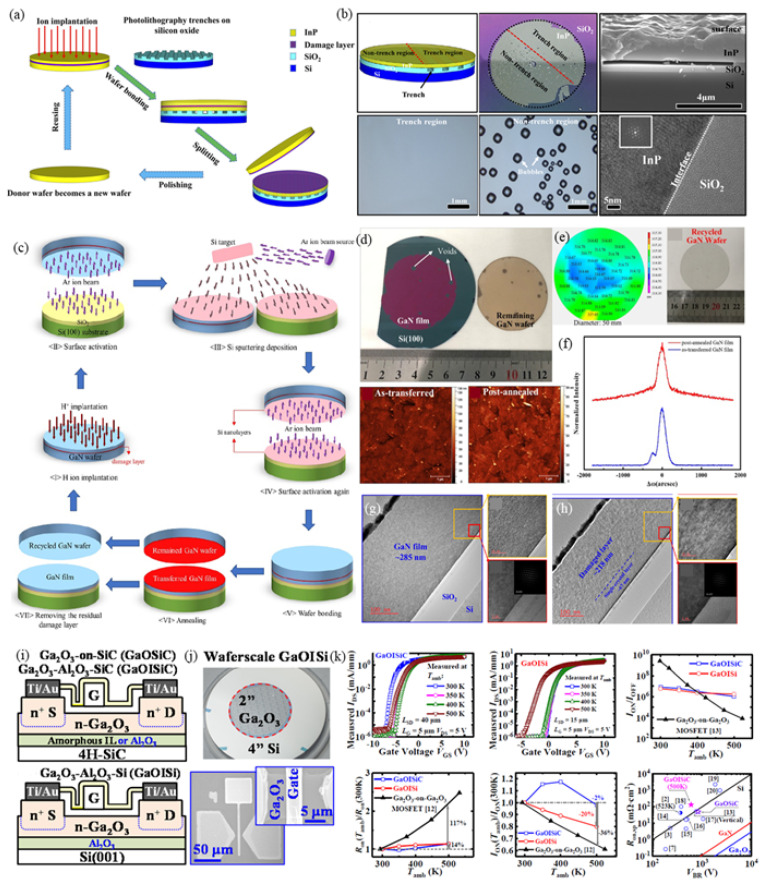
Heterogeneous bonding for III-V and wide bandgap semiconductor thin-film transfer onto Si substrate and their applications. (**a**) Schematic diagram of the modified Smart Cut^TM^ technology for InP thin-film transfer [88]. (**b**) Optical and SEM images for the transferred InP thin film onto Si substrate [88]. (**c**) Schematic diagram of the modified Smart Cut^TM^ technology for GaN thin-film transfer [89]. (**d**) Optical image of the transferred GaN thin film onto Si substrate [89]. (**e**) Thickness and AFM measurements for the transferred GaN thin film [89]. (**f**) XRD characterization for the GaN thin film before and after the annealing treatment [89]. (**g**,**h**) TEM observation for the bonding interface before and after the annealing treatment [89]. (**i**) Schematic diagram of the Ga_2_O_3_-based MOSFET [90]. (**j**) Optical image of the transferred wafer-level GaOISi and SEM image of the device structure [90]. (**k**) Performance characterizations of the Ga_2_O_3_-based MOSFET [90]. Reprinted with permission from ref. [88]. Copyright 2020 American Institute of Physics. Reprinted with permission from ref. [89]. Copyright 2020 Institute of Physics. Reprinted with permission from ref. [90]. Copyright 2020 Institute of Electrical and Electronics Engineers.

The InP thin-film transfer via wafer bonding is similar to the Smart Cut^TM^ technology. However, the generated gas originated from the InP/Si bonding will cause unavoidable bubbles in the interface, reducing the usage of integrated heterogeneous wafers. A modified method using surface trenches to eliminate interfacial voids has been proposed [88]. The schematic diagram of the modified Smart Cut^TM^ technology for InP thin-film transfer is shown in Figure 3a. Before the wafer bonding, trenches are patterned on the SiO_2_ layer using photolithography followed by the plasma etching process. By optimizing the parameter of trench space, the bubbles can be efficiently inhibited. To highlight the effect of trenches, half of the SiO_2_-on-Si wafer with channels and a half without channels are used for InP heterogeneous bonding. The bonding results after annealing are shown in Figure 3b. Experimental results show that there are many bubbles in the non-trench region, while the trench region is void-free. The interfacial SEM image shows that the InP/SiO_2_/Si bonding interface across the trench region is tight. TEM observation also confirms that the atomic bonding between InP and SiO_2_ has been achieved. Relying on this modified method, InP thin film with 2-in can be successfully transferred onto the SiO_2_-on-Si wafer. The whole area of the InP/SiO_2_/Si heterogeneous substrates is uniform, flat, and high-quality, can be used for practical device fabrication. We should note that the lateral outgassing surface trenches (LOTs) mechanism to facilitate the generated gas diffusion out of the bonding interface is a physical effect, only relying on the design of LOTs. This modified Smart Cut^TM^ technology might be a promising method to remove the bubbles at the bonding interface for all III-V compound semiconductors when integrated with the SiO_2_, SiO_2_-on Si, or Si substrates.

### 3.2. Wide Bandgap Semiconductor Thin-Film Transfer and Its Applications in MOSFETs

Gallium nitride (GaN) is an ideal material to apply in wireless communications and optical communications because it can operate in high power and frequency [91,92,93]. Thus, the heterogeneous integration of GaN with Si-CMOS substrate has attracted wide attention [94]. However, due to the significant lattice mismatch between GaN and Si, joining two kinds of materials into one system is challenging. Recently, direct epitaxy growth of GaN film on the (100)-oriented Si substrate has been reported. The generated GaN thin film is usually polycrystalline with a two-domain structure because of the asymmetric surface domains of the Si substrate. A modified surface activated bonding (SAB) method has been proposed to achieve the GaN thin film transfer combining with the Smart Cut^TM^ process [89]. The schematic diagram of the GaN thin-film transfer onto the Si substrate is shown in Figure 3c. Because of the high bonding strength of modified SAB, the GaN/SiO_2_/Si bonding interface can keep stable during the post-annealing to peel off the GaN thin film from the bulk GaN wafer. The optical image of the transferred GaN-on-insulator (GaNOI) wafer is shown in Figure 3d. The area ratio of the transferred thin film to the donor GaN wafer is more than 90%. A small number of bubbles are caused by the particles adsorbed on the wafer surfaces. The deep cleaning process can avoid this kind of defect. Comparing the surface roughness before and after annealing treatment, the RMS values are 6.81 nm and 6.95 nm, respectively, in the area of 5 μm × 5 μm, demonstrating the modified SAB method can obtain sufficient bonding strength to resist the interfacial stress. The thickness mapping of the transferred thin film shows that the thickness is nearly uniform in the whole wafer, as presented in Figure 3e. After griding and CMP for the remaining GaN wafer, the color recovered from golden brown to transparent. It indicates that the damaged layer formed during the peel-off process has been removed. XRD and TEM experiments also confirm that the high-quality GaN thin film transferred process has been achieved, as shown in Figure 3f–h.

In addition to GaN semiconductor, Ga_2_O_3_ with ultra-wide bandgap has been considered a promising material for next-generation power electronics [95,96,97,98,99,100,101,102]. Unlike the GaN thin-film transfer, the modified SAB process uses nanometer-scale Al_2_O_3_ as the intermediate layer to perform the Smart Cut^TM^ process [90]. The transferred Ga_2_O_3_/Si heterogeneous structure can be used for the fabrication of MOSFETs. The schematic structure of the proposed Ga_2_O_3_ MOSFET on (001)-oriented Si substrate is shown in Figure 3i. An optical image of the transferred wafer-level Ga_2_O_3_ thin film and SEM image of the MOSFET microstructure is presented in Figure 3j. Due to the high-quality bonding, the device structure has been well prepared. The integrated wafers also bring lots of benefits in performance, as shown in Figure 3k. A device *V_br_* above 600 V is achieved at 500 K, which is weakly dependent on the temperatures. It shows a significant improvement in thermal stability compared with the Ga_2_O_3_-on-SiC devices. Therefore, it proves that thin-film transfer via wafer bonding is a promising method to overcome the thermal limitation in Ga_2_O_3_ power electronic applications.

## 4. Heterogeneous Bonding for Wide-Bandgap Semiconductors Thin-Film Transfer onto SiC or Diamond Substrates for High Heat Dissipation

Although the heterogeneous integration of wide-bandgap semiconductors and Si-CMOS can bring many benefits, applications of GaN and Ga_2_O_3_ are mainly high-power or high-frequency electronic devices because of their excellent material properties [103,104]. However, the high channel temperatures induced by self-heating usually degrade the device’s performance and reliability. One of the most effective methods is to use the heat dissipation substrates to remove the heat in time. SiC is used for heat dissipation substrates for GaN due to its high thermal conductivity and small lattice mismatch with GaN [13,105,106,107,108,109]. Unfortunately, the existence of the thermal boundary conductance (TBC) between GaN and SiC limits heat transport, making significant errors in theoretical calculations and experiments [110,111]. The GaN can grow on the SiC substrate with the assistance of the AlN intermediate layer, which is vital to growing high-quality GaN thin film. The issue, such as dislocation density, usually appears, making the interface thermal resistance more serious. In addition to MBE and metal-organic chemical vapor deposition, wafer bonding technology is another feasible method for fabricating heterogeneous GaN/SiC structures. Compared with thin-film growth and deposition, wafer bonding has many advantages, such as insensitive to lattice mismatch, low temperatures, wafer-scale, etc. [112,113,114,115,116,117,118,119].

**Figure 4:**
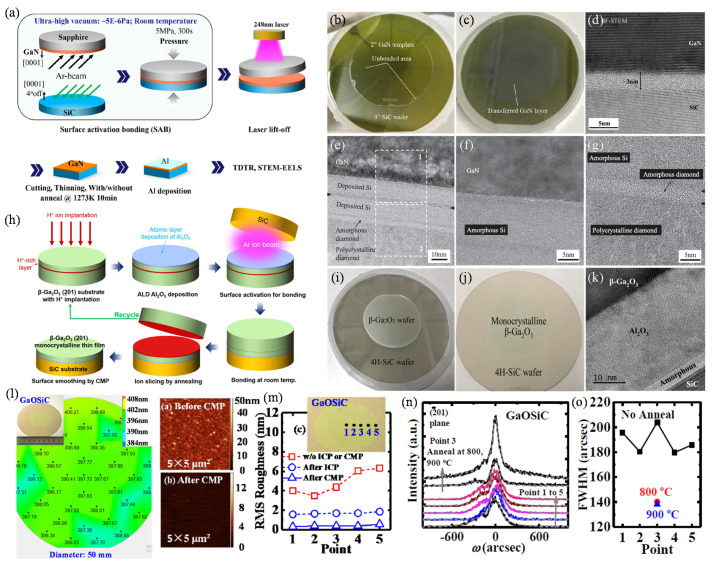
Heterogeneous bonding for wide-bandgap semiconductors thin-film transfer onto SiC or diamond substrates for high heat dissipation. (**a**) The schematic diagram for GaN thin-film transfer onto the SiC substrate via Si-modified SAB method [12]. (**b**) Optical image of the bonded GaN and SiC wafers via Si-modified SAB method [12]. (**c**) Optical image of the transferred GaN thin film onto SiC substrate [12]. (**d**–**g**) TEM images of the GaN/SiC bonding interface [12]. (**h**) The schematic diagram for Ga_2_O_3_ thin-film transfer onto the SiC substrate via Al_2_O_3_-modified SAB method [121]. (**i**) Optical image of the bonded Ga_2_O_3_ and SiC wafers via Al_2_O_3_-modified SAB method [121]. (**j**) Optical image of the transferred Ga_2_O_3_ thin film onto SiC substrate [121]. (**k**) TEM images of the Ga_2_O_3_/SiC bonding interface [121]. (**l**) Thickness and AFM measurements for the transferred Ga_2_O_3_ thin film onto SiC substrate [90]. (**m**–**o**) Material characterizations of the transferred Ga_2_O_3_ thin film [90]. Reprinted with permission from ref. [12]. Copyright 2019 American Chemical Society. Reprinted with permission from ref. [121]. Copyright 2020 American Chemical Society. Reprinted with permission from ref. [90]. Copyright 2020 Institute of Electrical and Electronics Engineers.

To achieve a reliable bonding between GaN and SiC, the SAB method has been used for direct wafer bonding [12]. Different from the Smart Cut^TM^ technology, the laser lift-off is used for the GaN thin-film transfer. The schematic diagram of the experimental process is shown in Figure 4a. The GaN is grown on the sapphire wafer and used as the donor substrate. After the GaN/SiC bonding, the 248 nm laser irradiates on the bonded wafer pair to make GaN and sapphire separate. Optical images of the bonded wafer pair and transferred GaN thin film are shown in Figure 4b and Figure 4c, respectively. Atomic bonding has been confirmed by TEM, and the bonding interface is only 3 nm, as shown in Figure 4d. This indicates that a high-quality GaN/SiC heterogeneous structure can be obtained by the SAB method combined with a laser lift-off process. Diamond is another popular substrate for thermal management [120]. The GaN thin film can also be transferred onto the diamond substrate via the bonding method [11]. Instead of the intermediate-free SAB method, nanometer-scale amorphous Si layers are used for the GaN/diamond bonding. The Si is sputtered to the GaN and diamond substrates before the surface activation. The followed processes are the same as the GaN thin-film transfer onto the SiC wafer. Atomic bonding between GaN and diamond has been realized, as shown in Figure 4e–g. The bonding interface is smooth and void-free, demonstrating the effectiveness of the GaN thin-film transfer to the heat sink substrates.

Not only limited to the GaN, the SAB method for the thin-film transfer is also suitable for the Ga_2_O_3_ [90,121,122]. The Ga_2_O_3_ thin-film transferred procedure is the same as the Smart Cut^TM^ process, as shown in Figure 4h. However, the bonding method is using nanometer-scale Al_2_O_3_ layers as the intermediate for the modified SAB method. Optical images of the Ga_2_O_3_/SiC bonded wafer pair and transferred Ga_2_O_3_ thin film are shown in Figure 4i and Figure 4j, respectively. TEM observation shows that atomic bonding between Ga_2_O_3_ and SiC has been achieved bridging by Al_2_O_3_, as presented in Figure 4k. The thickness mapping of the transferred thin film shows that the thickness is nearly uniform in the whole Ga_2_O_3_/SiC wafer, as presented in Figure 4l. The surface roughness can also satisfy the high requirements of device fabrication after a CMP surface treatment confirmed by multi-point testing. The RMS values are stable at ~0.5 nm with a measured area of 5 μm × 5 μm. After a 900 °C post-annealing treatment, the FWHM of XRD rocking curves for the Ga_2_O_3_/SiC heterogeneous wafer is reduced to 130 arcsec, demonstrating the high crystallinity of the Ga_2_O_3_ layer.

## 5. Piezoelectric Thin Transfer for the Self-Powered Implantable Electronics

Implantable electronics have important research significance for human health detection, disease diagnosis, and treatments. Several intelligent automatic devices have been applied in clinical practice to improve patients’ health. Although many self-powered devices, such as triboelectric- or electrochemical-based devices, can detect the human body’s dynamics, they cannot be applied in-vivo because of the limited biocompatibility of materials [29,123,124,125,126,127,128,129,130,131,132,133]. Among many kinds of materials, piezoelectric materials have always attracted much attention due to their mature preparation processes and high-efficiency responses to the deformation [134,135,136]. Although (K, Na)NbO_3_ (KNN) is more friendly to the human body and environment because it is lead-free, lead zirconate titanite (PZT) has more advantages in the performance and fabrication of implanted devices due to the higher piezoelectric effect [137,138,139,140,141,142,143,144]. Therefore, by transferring the PZT thin film to a flexible substrate with good biocompatibility, such as polydimethylsiloxane (PDMS) and polyimide (PI), it can be used for the fabrication of self-powered implantable electronics with different functions. Herein, methods for the piezoelectric thin film transfer onto PDMS and PI will be introduced.

**Figure 5:**
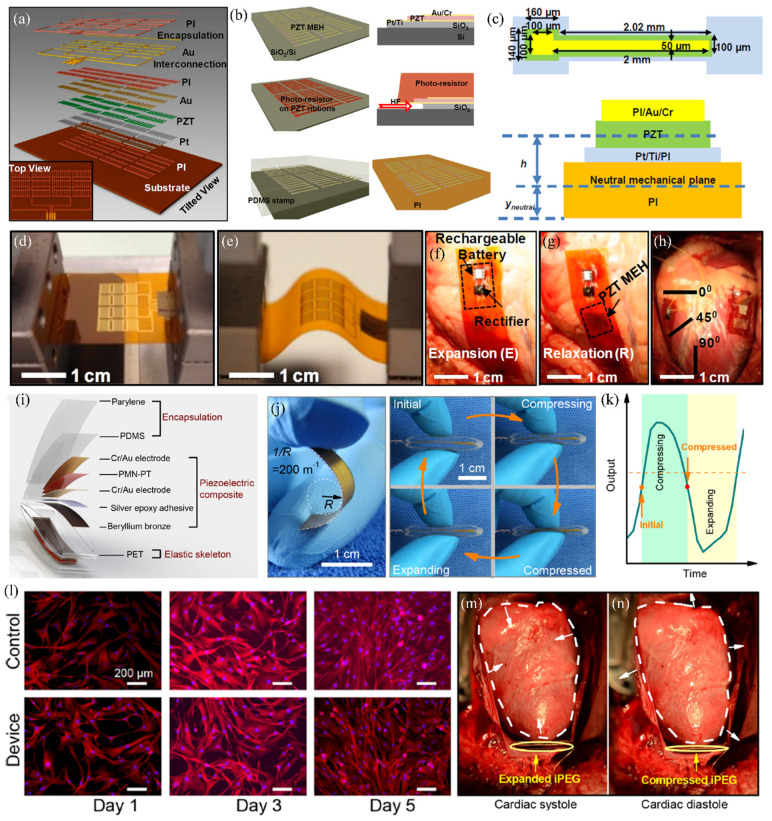
Piezoelectric thin transfer for the self-powered implantable electronics. (**a**) Exploded view and top view of the flexible MEH based on thin ribbons of PZT [145]. (**b**) Schematic illustration of procedures for fabricating a PZT MEH on a polyimide (PI) substrate [145]. (**c**) Top and cross-section view of a single PZT ribbon capacitor structure [145]. (**d**,**e**) Optical recordings of a PZT MEH clamped on a bending stage in flat and bent configurations [145]. (**f**,**g**) Images of PZT MEHs on the RV, LV, and free wall of a bovine heart [145]. (**h**) PZT MEH cointegrated with a rectifier and rechargeable battery [145]. (**i**) Exploded view of the detailed composited structures for the implantable piezoelectric generator [147]. (**j**,**k**) Piezoelectric composite bending by human fingers and corresponding output signals [147]. (**l**) Biocompatibility tests of the implantable piezoelectric generator using human pericardial fibroblasts [147]. (**m**,**n**) Optical images for the real-time monitoring of heart test using the proposed implantable device [147]. Reprinted with permission from ref. [145]. Copyright 2014 United States National Academy of Sciences. Reprinted with permission from ref. [147]. Copyright 2019 American Chemical Society.

The PZT thin film transfer onto different flexible substrates is based on the debonding method of hydrofluoric (HF) acid to remove the SiO_2_ layer [145]. The final exploded view, and top view of the flexible mechanical energy harvester (MEH) based on thin ribbons of PZT are shown in Figure 5a. The schematic illustration for the fabrication of MEH composite structures is displayed in Figure 5b. Firstly, the composite PZT with metal electrodes is deposited onto the SiO_2_-on-Si wafers by the CMOS-compatible process. The device structures have also been transferred at the same time. Secondly, the photoresist is spin-coated on the wafer surfaces to prevent the device structure from being corroded by subsequent HF solutions. After the immersion treatment, the deposited PZT thin film with photoresist protection will be separated from the SiO_2_-on-Si wafer. Finally, the functional thin film will be stamped by the PDMS onto the PI substrate for the implantable electronics. Based on this process and composite structures, implantable devices that enable high-efficiency mechanical-to-electrical energy conversion have been proposed. The device parameters and structures are shown in Figure 5c. With the excitation of bending treatment, the PZT thin film will generate an electrical signal responded to the deformation. Optical images of a PZT MEH clamped on a bending stage in flat and bent conditions are presented in Figure 5d and Figure 5e, respectively. Due to PI has good biocompatibility, such kind of device can be used for self-powered implant devices in the organism [146]. Photographs of PZT MEHs on the right ventricle (RV), left ventricle (LV), and free wall of a bovine heart are shown in Figure 5f–h. Different signal outputs will be produced under different monitoring systems. It demonstrates the effectiveness of PZT thin film transfer and the feasibility of biocompatible electronics relying on such composite structures.

The same process can also be used for the piezoelectric composite thin film transfer onto the PDMS substrate [147]. Recently, an implantable piezoelectric generator (iPEG) has been proposed to monitor and treat severe diseases. The device structures are shown in Figure 5i. It can be used for the battery-free implantable cardiac pacemaker. As shown in Figure 5j, different signal outputs will show up with different shape changes, as presented in Figure 5k. Therefore, information about heartbeats will be recorded and output as electrical signals.

Moreover, the biocompatibility of the iPEG has also been tested, as shown in Figure 5l. The fabricated composite device has almost no adverse effects on biological tissues. The live heartbeat test has also been performed, as shown in Figure 5m,n. The heart beating will compress the device to varying degrees to record information for the subsequent health monitoring and disease diagnosis. Since the device fabrication process is CMOS compatible, the device possesses excellent stability and can be applied for mass production.

## 6. LiNbO_3_ Thin-Film Transfer for High-Performance Electro-Optical Modulators

**Figure 6:**
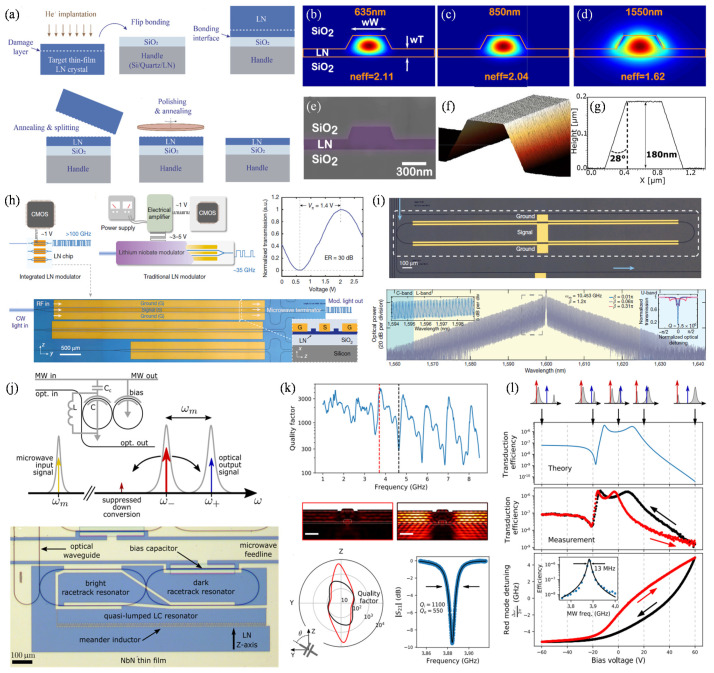
LiNbO_3_ thin-film transfer for high-performance electro-optical modulators. (**a**) Schematic diagram of the LiNbO_3_ thin-film transfer for the fabrication of LNOI wafer [148]. (**b**–**d**) Finite element simulation of the TE_00_ waveguide mode near the wavelengths of 635 nm, 850 nm, and 1550 nm [22]. (**e**) SEM image of the cross-sectional waveguide [22]. (**f**,**g**) Surface morphology and surface profile of the LiNbO_3_ waveguide [22]. (**h**) Schematic diagram and optical images of the nanophotonics LiNbO_3_ modulator compatible with CMOS drive voltages [21]. (**i**) Optical image of the fabricated LiNbO_3_-based comb generator [20]. (**j**) Schematic diagram and optical image of the designed superconducting cavity electro-optic transducer based on the LNOI platform [149]. (**k**) Performance test of the piezoelectric loss in LiNbO_3_ [149]. (**l**) Performance measurements for the detuning dependence of microwave-to-optical photon transduction [149]. Reprinted with permission from ref. [148]. Copyright 2020 World Scientific. Reprinted with permission from ref. [22]. Copyright 2019. The Optical Society. Reprinted with permission from ref. [21]. Copyright 2018 Springer Nature. Reprinted with permission from ref. [20]. Copyright 2019 Springer Nature. Reprinted with permission from ref. [149]. Copyright 2020 The Optical Society.

LiNbO_3_ is a popular material due to its excellent piezoelectric property, electro-optic and nonlinear optical effects. However, traditional bulk LiNbO_3_-based optical devices suffer from large volume, expensive, low bandwidth, and high operating voltages. To overcome this problem, thin-film LiNbO_3_ is a good choice. Currently, there are already several kinds of commercial LiNbO_3_ thin-film wafers, such as LiNbO_3_-on-Si, LiNbO_3_-on-sapphire, LiNbO_3_-on-glass, etc. Fabrication of the above LiNbO_3_-based heterogeneous wafers is based on the Smart Cut^TM^ technology [148]. The process is shown in Figure 6a. He^+^ implantation is performed to create a damage layer in the LiNbO_3_ sub-surface. After surface activation, the activated surfaces of LiNbO_3_ and handle substrate are contacted to form the pre-bonded pair. With an annealing treatment, the donor LiNbO_3_ wafer is cracked from the damage layer. At the same time, the bonding interface between the LiNbO_3_ thin film and handle wafer will be strengthened because of the higher temperatures. After the CMP process, heterogeneous wafers can be used for device fabrication. However, LiNbO_3_ is a kind of chemically inert material. The large propagation loss caused by the rough sidewalls is due to the imperfect fabrication. A rib configuration for the LiNbO_3_-based optical devices has been proposed to better confine the waveguide light, as shown in Figure 6b–g [22]. Electro-optic (EO) modulators and EO comb generators are essential building blocks for modern telecommunication networks, quantum photonics, and microwave-photonic systems. The excellent properties of LiNbO_3_ can satisfy the requirements of high-performance EO devices. Recently, a 50:50 Y-junction Mach-Zehnder interferometer and an EO comb generator have been proposed, as shown in Figure 6h and Figure 6i, respectively [20,21]. The EO modulator can work at a CMOS-compatible voltage (1.4 V) with high bandwidth. The transmission speed of data can reach 210 Gbit/s. As for the LiNbO_3_-based EO comb generator, the frequency range covering the entire telecommunication L-band has been achieved. Both demos demonstrate that the LiNbO_3_-based EO devices have huge potential in applying 5G and even the next generation of communication technology.

Transducers are promising platforms that may enable large-scale quantum networks because of the direct conversion, low-noise operation, and large bandwidth. A superconducting cavity EO transducer based on the LiNbO_3_ thin film has been proposed [149]. The schematic diagram and working mechanism of the designed device are shown in Figure 6j. The optical pump signal can be tuned to the red optical at a specific frequency because two microring resonators are evanescently coupled. The simulated and measured results for the piezoelectric loss in LiNbO_3_ are shown in Figure 6k. It can be seen that frequencies near the bulk acoustic wave modes present strong loss, while low loss is observed for frequencies far detuned from the bulk acoustic modes. For the detuning dependence of microwave-to-optical photon transduction, the results are shown in Figure 6l. The experimental measurements are in good agreement with the theory. Therefore, the LiNbO_3_-based EO transducer shows system simplicity and low-noise operation, showing opportunities for the on-chip filter integration and further improvement of the transduction efficiency.

## 7. Si-on-CaF_2_ Platform Fabrication for MIR Sensors

There are many fingerprints for gases, liquids, and biomolecules in the MIR wavelength [150,151,152,153,154,155,156,157,158,159]. Correspondingly, the working wavelengths of many sensors are designed in the range of MIR [160,161,162,163,164,165,166,167,168,169,170]. In addition to microelectronics, Si is also one of the most popular materials in photonics [171,172]. Due to its high refractive index, excellent transparent properties in the spectral range from 1.2 μm to 8.0 μm, and high-quality commercial product of SOI platform, all kinds of Si-based sensors have been well developed. Although SOI has good confinement for light due to the significant differences in the refractive index between Si (*n* = 3.48) and SiO_2_ (*n* = 1.40), the existence of SiO_2_ limits the operating wavelength range of Si photonics because of the light absorption. To make Si-based devices have better performance when the working wavelength is larger than 4 μm, transferring Si thin film to infrared substrates with lower refractive indexes, such as sapphire, MgF_2_, CaF_2_, and BaF_2_, is a good choice.

**Figure 7:**
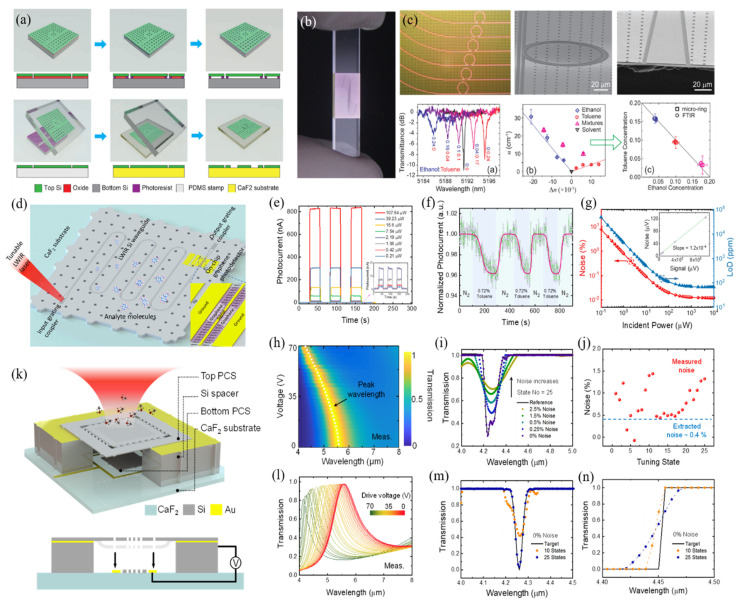
Si thin-film transfer onto the CaF_2_ substrate for the MIR sensing applications. (**a**) Schematic illustration of the Si thin film transfer process [173]. (**b**) Optical image of the fabricated Si photonic device on a CaF_2_ substrate [173]. (**c**) Optical and SEM images of the fabricated microring sensors with their performance test [173]. (**d**) Schematic illustration of the heterogeneously integrated graphene/Si/halide Si-based MIR photodetectors [174]. (**e**–**g**) Dynamic on-chip sensing demonstration [174]. (**h**–**j**) Simulation and experimental results for the spectral tuning and gas sensing [175]. (**k**) Schematic diagram of the MEMS-based tuning optical gas sensor [175]. (**l**–**n**) Simulation and experimental results for the spectral tuning and gas sensing. Reprinted with permission from ref. [173]. Copyright 2014 American Chemical Society.Reprinted with permission from ref. [174]. Copyright 2021 American Chemical Society. Reprinted with permission from ref. [175]. Copyright 2021 Institute of Electrical and Electronics Engineers.

The primary fabrication process for developed Si thin-film transfers onto the CaF_2_ substrate is shown in Figure 7a [173]. The SOI platform is composed of a top Si layer, buried silicon dioxide (BOX) layer, and Si substrate, the donor wafer for the Si thin film. Before the thin-film transferred process, an array of holes is patterned on the top Si layer using photolithography and dry etching methods. These holes are used for the hydrofluoric acid solution to access the BOX and removing the SiO_2_ layer. Due to the existence of photoresist, the underlying cured and baked photoresist become pedestals to support the top Si layer on the supporting substrate after completely releasing the BOX. Subsequently, a polydimethylsiloxane (PDMS) film with a thickness of several micrometers is used to stick on the surface of the released top Si thin film. We should note that the contact between PDMS and Si thin film mainly depends on the adhesion force, which can be regarded as temporary bonding, but the bonding strength is stronger than the fracture strength of the photoresist. Therefore, the Si membrane can be peeled off from the SOI platform. After that, the Si/PDMS composite is pressed on the receiving CaF_2_ substrate, and PDMS is slowly removed. Consequently, the Si thin film is bonded onto the CaF_2_ substrate with the assistance of surface forces. Although the bonding of Si and CaF_2_ is under the effects of van der Waals and other temporary bonds, experiments have verified that the substrate can withstand the erosion of chemical solutions.

The above-mentioned method is capable of transferring Si thin film with an area of several square centimeters. Based on this process, the first Si photonic device integrated on the CaF_2_ substrate has been fabricated and published [173]. Figure 7b shows the optical image of the microring arrays fabricated on the transferred Si-on-CaF_2_ substrate. It can be seen that the surface is flat and smooth. The microstructures of the Si microring resonators coupled with bus waveguides are shown in Figure 7c. The waveguides are inversely tapered toward the edge of the chip to facilitate butt-coupling with infrared fibers. Due to the working wavelengths that can be designed beyond 4.0 μm, the mixed ethanol and toluene ratio can be distinguished. Moreover, the high-performance MIR resonator based on this transferred platform can be used to analyze organic chemicals with a limit of detection of less than 0.1 ng.

The heterogeneous integration of waveguide photodetectors towards chip-scale zero-bias long-wave infrared (LWIR) spectroscopic sensing has also been demonstrated [174]. The schematic diagram of the proposed on-chip LWIR spectroscopic sensor is shown in Figure 7d. The graphene overlays the waveguide as a photodetector. When the light with different wavelengths propagates along the multiple waveguide channels, the analytes will extensively interact with the evanescent field. Then, both light intensity and photoresponse are decreased at the specific absorption peaks. The measured device performances are shown in Figure 7e–g. Experimental results show that the photocurrent changes linearly with the increased light power. In addition to the passive device, the functional system design, such as MEMS-based tuning spectra, is shown in Figure 7k [175]. To avoid the limitation of stress in device fabrication, double layers of thin PCS reflectors are transferred to form the Fabry–Pérot filter (FPF). By applying different voltages, the transferred Si thin film can be deformed to different degrees. The simulation and experimental results show that the spectra can be tuned with different voltages, as presented in Figure 7h–n. When the applied voltage is 70 V, the peak position is decreased from 5.61 μm to 4.13 μm. By reconstructing the tunable spectra, this kind of device can be used for sensing applications. As a proof of concept, CO_2_ molecules have been introduced for measurement. A dip recovering with a full width at half maximum of 170 nm at 4.26 μm has been observed. More high-performance passive or active miniaturized MIR devices will be developed based on the technology of Si thin-film transfer onto the CaF_2_ substrate.

## 8. High-Quality Thin Film Obtained via the Debonding Method for Flexible Electronics

Flexible and wearable electronics are an inevitable trend in the development of real-time and dynamic monitoring for human health since the thin-film electronics can offer better performance than traditional bulk systems, being lightweight, low-cost, and visible [17,23,24,28,176,177,178,179,180]. Although significant progress in the fabrication of thin-film materials and optimization of structure design, the realization for high-performance thin-film electronics compatible with a wafer-level batch process is still limited [181,182,183,184,185,186,187,188]. Thus, the development of transfer printing technology in the wafer-level for thin-film micro/nanoelectronics is important for high-yield production. In the above sections, we have introduced several thin-film transferred technologies combined with bonding and debonding methods based on the Smart Cut^TM^ technology. In such situations, bonding is more critical to achieving reliable fabrication for electronic and photonic devices. However, for the thin-film transfer printing process at the wafer level, debonding is more critical to obtain flexible substrates with large size and fewer surface damages. In this part, we introduce two kinds of technologies (i.e., SiO_2_ layer released and water-induced layer separation) to fabricate high-quality thin-film substrates.

**Figure 8:**
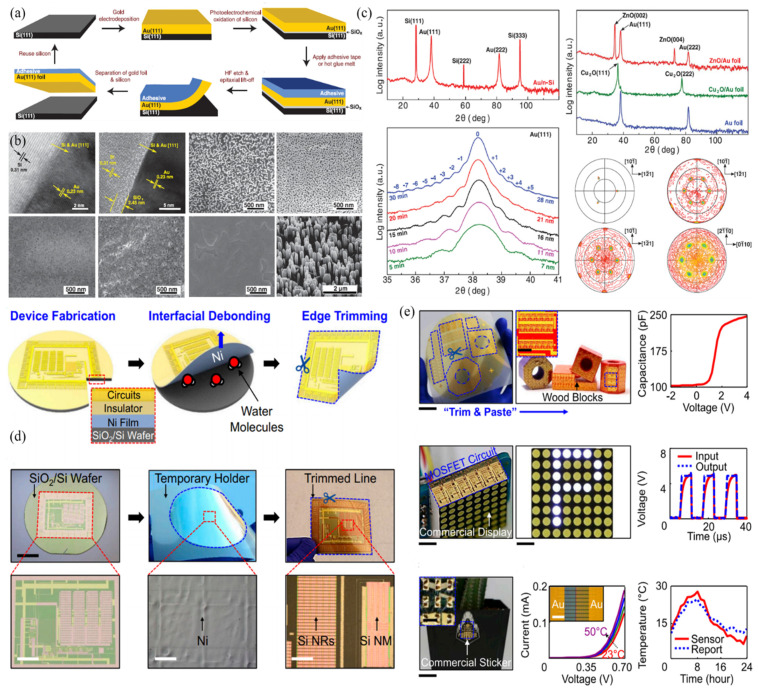
The high-quality thin film is obtained by the debonding method for flexible electronics. (**a**) The schematic diagram for the epitaxial lift-off of single-crystal Au foil by the release of SiO_2_ layer [189]. (**b**) Comparison for the high-resolution TEM studies of the epitaxial grown and transferred Au film with the application of Cu_2_O and ZnO growth [189]. (**c**) In-plane and out-of-plane orientation studies by the X-ray diffraction and pole figure for the transferred Au foil [189]. (**d**) Schematic illustration of the thin-film transfer for large-area nanoelectronics based on the SiO_2_ layer releasable method [190]. (**e**) Device fabrication on the transferred thin film and its performance characterizations [190]. Reprinted with permission from ref. [189]. Copyright 2017 American Association for the Advancement of Science. Reprinted with permission from ref. [190]. Copyright 20118 United States National Academy of Sciences.

Nano-thickness metal films possess excellent properties in optical transmittance, flexibility, and sheet resistance. Most of them are generated on the substrates by evaporation, sputtering, or other deposition methods, making the thin films polycrystalline. However, the grain boundaries often make electronics suffer from electron-hole recombination, which will degrade their performance. To overcome this problem, introducing a single-crystal Au film on the Si surface as a substrate for epitaxial growth of other single-crystal metal thin films has been developed [189]. The schematic diagram for the Au foil transferred process is shown in Figure 8a. Wafer-scale single-crystal Au film is grown on the Si substrate by the epitaxial growth method. With the help of light irradiation, a sacrificial SiO_x_ layer sandwiched by the Au foil and Si template is generated due to the photoelectrochemically oxidizing. Then, a polymer adhesive is attached to the Au surface for the subsequent thin-film separation. By immersing the composite layer in the hydrofluoric acid solution, the SiO_x_ layer is removed to achieve the peeling. The interfacial microstructures in each step are shown in Figure 8b. Both single-crystal Au film and oxidized SiO_2_ have been confirmed by TEM. A 28 nm-thickness Au foil with a sheet resistance of 7 ohms per square shows only a 4% increase after 4000 bending cycles. Besides, the grown ZnO nanowires also show flexibility with the nanowires intact up to 500 bending cycles. X-ray diffraction and pole figures have been used to study the in-plane and out-of-plane orientation, as shown in Figure 8c. High-quality single-crystal metal films have been confirmed. Therefore, this process provides a universal method for the production of ordered substrates for flexible electronics.

The schematic illustrations of critical steps for the thin film transfer based on the water-induced layer separation are shown in Figure 8d [190]. Nanometer-thickness Ni and dilute polyimide are deposited on a SiO_2_/Si wafer to serve as the separation and glue layer, respectively. With water molecules’ assistance, the glue layer dissolves, and the device layer peels off from the substrate. Additionally, the SiO_2_/Si wafer can be repeatedly used. Optical images of the n-doped Si thin-film capacitor arrays on a thermally releasable tape, and device performance test are shown in Figure 8e. Due to the robust structures, the transferred flexible electronics can be pasted on objects with different shapes. Moreover, the preset functions of the device can be well realized. This debonding method can be effectively performed in wafer-recyclable, environmentally friendly transfer printing for large-scale thin-film nanoelectronics.

## 9. Concluding Remarks and Perspectives

In the past few decades, wafer bonding technology has made remarkable achievements in SiP and the fabrication of heterogeneous structures for electronics. Although it is only one step in developing various platforms, wafer bonding is one of the most essential and indispensable steps in obtaining reliable interfaces among device layers and substrates. Thanks to the extraordinary performance by thin-film, the micro/nano devices show great enhancement in many applications of electronics devices, optoelectronics devices, infrared nanophotonic sensors, and wearable sensors compared with their counterpart made by bulk materials. Although the cost for the wafer increases at the current stage, the gain of device performance is requesting the industry to lower down the cost by developing mature processes for mass production. With the rapid development of 5G technology and IoTs, the fabrication of new heterostructures suitable for various functional devices ushered in both challenges and opportunities. Compared with the developments of bonding methods for semiconductors, more work needs to contribute to infrared material bonding. With the emerging rise of metasurfaces in molecular sensing, more heterogeneous composites for infrared materials need to be developed to satisfy the theoretical research and experimental requirements. In the future, wearable and multifunctional system-level integration combined with extensive data analysis and artificial intelligence is an inevitable trend for sensing applications. Wafer bonding is one of the feasible and effective methods for such a large and complex heterogeneous integration.
